# The association of an elevated Th/Ts ratio and lupus anticoagulant with symptomatic osteonecrosis in systemic lupus erythematosus patients

**DOI:** 10.3389/fimmu.2024.1288234

**Published:** 2024-02-07

**Authors:** Ruihong Hou, Jiamin Lei, Dengfeng Xue, Yukai Jing, Liangyu Mi, Qianyu Guo, Ke Xu, Liyun Zhang

**Affiliations:** ^1^ Department of Rheumatology, Third Hospital of Shanxi Medical University, Shanxi Bethune Hospital, Shanxi Academy of Medical Sciences, Tongji Shanxi Hospital, Tongji Medical College, Huazhong University of Science and Technology, Taiyuan, China; ^2^ Department of Galactophore Surgery, The Second Hospital of Shanxi Medical University, Taiyuan, China; ^3^ Department of Clinical Laboratory, Third Hospital of Shanxi Medical University, Shanxi Bethune Hospital, Shanxi Academy of Medical Sciences, Tongji Shanxi Hospital, Tongji Medical College, Huazhong University of Science and Technology, Taiyuan, China

**Keywords:** risk factors, symptomatic osteonecrosis, systemic lupus erythematosus, lymphocyte, lupus anticoagulant

## Abstract

**Objective:**

This study aimed to assess the risk factors for symptomatic osteonecrosis (ON) in systemic lupus erythematosus (SLE) and identify clinical characteristics and laboratory markers for predicting symptomatic ON occurrence in SLE patients.

**Methods:**

Seventy (6.0%) of 1175 SLE patients diagnosed with symptomatic ON were included in this study. An equal number of SLE patients without symptomatic ON, matched in terms of age and gender, were enrolled in the control group. Clinical symptoms, routine laboratory examinations, lymphocyte subsets, and treatments of these patients were retrospectively reviewed and compared between the two groups. Logistic regression analysis was employed to identify risk factors associated with symptomatic ON in SLE.

**Results:**

Among the 70 cases in the symptomatic ON group, 62 (88.6%) patients experienced femoral head necrosis, with bilateral involvement observed in 58 patients. Bone pain was reported in 32 cases (51.6%), and 19 cases (30.6%) presented with multiple symptoms. Univariate analysis revealed significant differences between the two groups in various factors, including disease duration (months), cumulative steroid exposure time, history of thrombosis, neurological involvement, the number of affected organs, myalgia/myasthenia, and the use of medications such as glucocorticoids, immunosuppressants, aspirin, and statins (P<0.05). Moreover, lupus anticoagulant (LA) levels were significantly higher in the symptomatic ON group than in the control group (P<0.05). Furthermore, notable distinctions were observed in peripheral blood immune cells, including an elevated white blood cell count (WBC), a decreased percentage of Ts cells (CD3+CD8+), and an elevated Th/Ts ratio. Logistic regression analysis revealed that a history of thrombosis, LA positivity, and an elevated Th/Ts ratio remained positive factors associated with symptomatic ON (P<0.05).

**Conclusion:**

Decreased Ts cells and changes in the T lymphocyte subset play an important regulatory role in the development of symptomatic ON. A history of thrombosis and LA are associated with an increased probability of symptomatic ON in SLE and may serve as potential predictors.

## 1 Introduction

Systemic lupus erythematosus (SLE) is a chronic autoimmune disease that can impact multiple organs and tissues, including the joints and skeletal system (1). SLE complicated with osteonecrosis (ON) is a rare yet severe complication. Reports indicate that the risk of ON in SLE patients varies from 3% to 44%, with an asymptomatic ON incidence of 40% (2–5). ON is a severe condition that leads to significant pain, joint surface collapse, and eventual physical disability. However, ON may be asymptomatic in its early stages. By the time clinical symptoms manifest, ON has typically reached an advanced and irreversible stage. Early diagnosis and timely treatment are crucial before symptoms manifest.

The pathophysiology of ON remains incompletely understood and is likely multifactorial, involving glucocorticoid use (GC), metabolic factors, genetic predisposition, and other influences on blood supply (6). Although GC therapy is commonly linked to increased ON risk in SLE patients, recent evidence challenges its sole responsibility (7). Studies show conflicting findings, suggesting a negative association between ON and GC therapy, and ON occurrence in GC-untreated SLE patients (8–10). Notably, among GC-treated autoimmune disorders, SLE patients exhibit the highest ON incidence. Factors like vasculitis, Raynaud’s phenomenon, and organ involvement in SLE have been linked to ON through mechanisms such as intraosseous vascular thrombosis (11, 12). Additionally, the role of anti-phospholipid antibodies (aPLs), known for their thrombosis-inducing potential, remains debated in ON development (10, 13).

Research also indicates that abnormal immune system activation and inflammatory responses in SLE patients might be primary factors in ON development. Factors such as immune complex deposition, chemokine receptors, regulatory cytokines and transcription factors can disrupt normal bone tissue function, resulting in the occurrence of ON (14). Previous studies have revealed heightened active T/Th1 and B cell levels in ON patients, producing receptor activators of nuclear factor kappa-B ligand (RANKL) and osteoprotegerin (OPG). Notably, CD4+ Th17 cells generate substantial active RANKL, tumor necrosis factor-a (TNF-a), interleukin-17 (IL-17), and IL-23 cytokine levels. Regulatory T (Treg) cells release anti-inflammatory cytokines, such as IL-4, IL-10, and transforming growth factor beta (TGF-β), inhibiting osteoclast formation (13). However, limited research exists on immune and immunomodulatory cell roles in SLE patients with ON.

Although some advances have been made in the research on SLE with ON, there are still challenges and unresolved issues. This study aims to analyze risk factors in SLE patients with symptomatic ON, particularly lymphocyte subsets, to identify new risk factors and enhance patient outcomes.

## 2 Methods

### 2.1 Study subjects

This retrospective study was based on inpatients diagnosed with SLE at the Shanxi Bethune Hospital between January 2012 and December 2022. All patients diagnosed with SLE met the classification criteria established by the Systemic Lupus International Collaborating Clinics (SLICC) in 2009. Their medical records were reviewed to identify patients with symptomatic ON. Among all 1175 patients, a total of 70 cases were diagnosed with SLE complicated with symptomatic ON, resulting in an incidence rate of 6.0%. To compare the clinical characteristics of SLE with symptomatic ON and analyze risk factors, 70 SLE patients without symptomatic ON were randomly matched in a 1:1 ratio based on age and gender during the same period. The median ages of the two groups at the time of study recruitment were 33 (27.75-44) and 32 (23–52) years, respectively. Ethical approval for the study was obtained from the Shanxi Bethune Hospital’s ethical committee.

### 2.2 Identification of symptomatic ON

The diagnosis of symptomatic ON was based on a combination of clinical evaluation and imaging studies. Most patients have clinical manifestations such as joint pain, movement restriction, and radiographic evidence, especially magnetic resonance imaging (MRI). MRI was performed on hips and knee joints with a high incidence of ON (15). These characteristic radiological features include irregular abnormal signal changes, characterized by prolonged T1 and T2 signals, or T1-weighted imaging (T1WI) showing low signals, with the inner layer of T2-weighted imaging (T2WI) displaying high signals and the outer layer showing low signals. Alternatively, the T1WI may exhibit a low-high-low signal pattern from the inner to outer layers, while the T2WI shows a high-low-high signal pattern. Additionally, the fat-suppressed sequence demonstrates a high-low-high signal pattern (16). The diagnoses of symptomatic ON were made by a team of experienced radiologists. The absence of osteonecrosis was determined based on clinical assessments and available diagnostic records. Consequently, asymptomatic osteonecrosis may be included in the control group. Patients with concurrent malignancy, a history of oral contraceptive use, or a history of trauma were excluded from the study.

### 2.3 Data collection

Comprehensive clinical data, including demographic characteristics, clinical manifestations, complications, treatments, and laboratory indicators, were gathered for all patients during their hospitalization. All information was retrieved from the patient’s medical records. The general characteristics included gender, age, body mass index (BMI), age of SLE onset (years), duration of SLE (months), the SLE Disease Activity Index (SLEDAI-2K), and history of thrombosis. The age matching for the two groups at enrollment followed a standard of plus or minus 5 years. The duration of SLE was calculated from the time of SLE onset to the time of the patient’s admission to the hospital. Organ involvement was collected, encompassing the skin, joints, myositis, kidneys, hematological systems, and the nervous system. Treatment strategies, including glucocorticoids, immunosuppressants (hydroxychloroquine, cyclophosphamide, mycophenolate mofetil, and azathioprine), aspirin, anticoagulants, calcium, and statins, were recorded. A daily dose of GC prednisone equivalent to ≥250 mg was defined as high-dose steroid pulse therapy. The cumulative hormone use time (months) refers to the duration from initiating hormone use to the patient’s admission.

Laboratory indexes included blood routine, erythrocyte sedimentation rate (ESR), complement, lymphocyte subsets, and autoantibody profiles. The analysis encompassed both absolute counts and proportions of lymphocyte subsets, comprising total T cells (CD3+), Th cells (CD3+CD4+), Ts cells (CD3+CD8+), B cells (CD3-CD19+), and natural killer (NK) cells (CD3-CD56+). Autoantibody profiles included antinuclear antibodies (ANA), anti-double stranded DNA antibodies (anti-dsDNA), anti-nucleosome antibodies, anti-histone antibodies, lupus anticoagulant (LA), anticardiolipin antibodies (aCL), and anti-β2GP-I antibodies. For anticardiolipin antibodies (aCL) and anti-β2GP-I antibodies, IgG, IgA, and IgM subtypes were all tested, with any one positive result considered positive.

### 2.4 Statistical analyses

Statistical analysis was performed using SPSS 26.0. The normality of continuous variables was assessed using the Shapiro–Wilk and Kolmogorov–Smirnov tests. Normally distributed continuous variables were presented as mean ± standard deviation and analyzed using independent sample t-tests. Non-normally distributed continuous variables were presented as median (interquartile and range) and analyzed using rank-sum tests. Categorical data were expressed as numbers (percentages) and analyzed using chi-square tests. For significant results in the univariate analysis, logistic multiple regression analysis was conducted to identify risk factors and protective factors influencing symptomatic ON. A significance level of P<0.05 was adopted for statistical significance.

## 3 Results

### 3.1 General information

In our study, 70 SLE patients (6.0%) were diagnosed with symptomatic ON. Additionally, we included 70 SLE patients without symptomatic ON. Among the SLE patients with symptomatic ON, 59 females and 11 males resulted in a female-to-male ratio of 5.36. The mean age of SLE onset was 29.36 ± 12.14 years in the symptomatic ON group and 32.26 ± 15.94 years in the control group. The average age at symptomatic ON onset was 32.73 ± 12.15 years for the symptomatic ON group. The duration of SLE was 76.21 ± 63.31 months for the symptomatic ON group and 52.11 ± 67.64 months for the control group (p<0.05). The time span between SLE onset and symptomatic ON occurrence ranged from 0 to 288 months, with a median of 60 months. The prevalence of a history of thrombosis was significantly higher in the symptomatic ON group than in the control group (P<0.05). No statistically significant differences were observed in terms of gender, BMI, age of SLE onset, and SLEDAI-2K scores (P>0.05) (**Table 1**).

**Table 1:** Demographic and clinical characteristics of SLE patients with or without symptomatic ON.

	SLE with symptomatic ON (n=70)	SLE without symptomatic ON (n=70)	*P*
General information			
Female	59 (84.3%)	62(88.5%)	0.459
BMI	23.5 ± 4.61	22.7 ± 4.02	0.331
Age (years)	33 (27.75-44)	32 (23–52)	0.638
Age of SLE onset (years)	29.36 ± 12.14	32.26 ± 15.94	0.228
Duration of SLE (months)	76.21 ± 63.31	52.11 ± 67.64	0.031*
SLEDAI-2K	8.5 (4.25-11.5)	9 (6-11.5)	0.659
History of thrombosis	10 (14.3%)	2 (2.9%)	0.017*
Organ involvement (n/%)			
Erythema	36 (51.4%)	24 (34.3%)	0.060
Joint pain	50 (71.4%)	41 (58.6%)	0.111
Myalgia/myasthenia	11 (15.7%)	3 (4.2%)	0.041*
Raynaud’s phenomenon	12 (17.1%)	14 (20.0%)	0.664
Renal involvement	42 (60.0%)	44 (62.9%)	0.728
Hematological involvement	55 (78.6%)	51 (72.9%)	0.430
Neurological involvement	24 (34.3%)	13 (18.6%)	0.035*
≥2 organs involvement	54 (77.1%)	35 (50%)	0.001*
Treatment			
Glucocorticoids	69 (98.6%)	63 (90.0%)	0.029*
High-dose steroid pulse therapy (>250mg/d)	15 (21.4%)	8 (11.4%)	0.110
Cumulative hormone use time (months)	55 (28.5-98.3)	14 (0-74.0)	0.001*
Immunosuppressants	64 (91.4%)	45 (64.3%)	<0.001*
Aspirin	34 (48.6%)	13 (18.6%)	<0.001*
Anticoagulants	11 (15.7%)	9 (12.9%)	0.629
Vitamin D	63 (90.0%)	61 (87.1%)	0.595
Calcium	63 (90.0%)	59 (84.3%)	0.313
Statins	24 (34.3%)	5 (7.1%)	<0.001*

Data are mean ± SD, median (IQR), or n (%). *Bold values indicate statistical significance (p<0.05).

### 3.2 Articular manifestations in SLE with symptomatic ON

Among the ON patients in this study, 62 (88.6%) exhibited femoral head necrosis, with bilateral involvement in 58 cases. Seventeen (24.3%) patients had knee necrosis. A total of 57 (81.4%) patients displayed an involvement of one to two bones, while the other 13 (18.6%) patients had an involvement of three or more bones. Among these patients, 32 (51.6%) experienced pain and 19 (30.6%) reported a combination of multiple symptoms.

### 3.3 Comparison of organ involvement and treatment between the two groups

As detailed in **Table 1**, significant statistical differences between the two groups were observed regarding myalgia/myasthenia and neurological involvement (P<0.05). The symptomatic ON group also exhibited a higher organ involvement incidence than the control group (P<0.05). Analysis of treatment strategies revealed a higher utilization rate of glucocorticoids, hydroxychloroquine, immunosuppressive agents, aspirin, and statins in the symptomatic ON group (P<0.05). Moreover, cumulative hormone use time significantly differed between the two groups. However, there were no notable distinctions in high-dose steroid pulse therapy, anticoagulants, and calcium use.

### 3.4 Comparison of laboratory findings between the two groups

Comparing the symptomatic ON patients with the control group, statistically significant increases were observed in white blood cell count (WBC), while complement C3 levels exhibited a decrease in the symptomatic ON group (P<0.05). The absolute Ts cell count did not significantly differ between the two groups regarding lymphocyte subsets. Yet, the percentage of Ts cells was lower in the symptomatic ON patients than in the control patients (P<0.05). Conversely, the Th/Ts ratio was notably higher in the symptomatic ON patients. Additionally, percentages of total T, Th, and B cells were elevated in the symptomatic ON group, but without significant statistical differences (**Table 2**). For SLE-related autoantibodies and aPLs, the percentage of LA positivity was markedly higher in the symptomatic ON patients (P<0.05), while other autoantibodies showed no significant variations between SLE patients with and without symptomatic ON.

**Table 2:** Laboratory findings of SLE patients with or without symptomatic ON.

	SLE with symptomatic ON (n=70)	SLE without symptomatic ON (n=70)	*P*
WBC (×10^9^/L)	6.85 ± 0.48	5.48 ± 0.44	0.010*
RBC (×10^9^/L)	3.76 ± 0.87	3.66 ± 0.85	0.508
Platelet (×10^9^/L)	183.48 ± 90.95	155.52 ± 86.64	0.076
HGB(g/L)	110.36 ± 24.24	111.39 ± 24.54	0.812
ESR(mm/h)	37 (18.25-60)	35 (17.75-54.5)	0.857
C3(g/L)	0.63 ± 0.26	0.76 ± 0.30	0.023*
C4(g/L)	0.14 ± 0.09	0.12 ± 0.07	0.224
Lymphocyte subset			
Total T %	78.71 ± 10.71	74.19 ± 12.94	0.059
Total T (cells/μL)	752.50 (436.50-1189.80)	609.50 (377.25-991.50)	0.178
Th %	32.90 ± 11.14	31.24 ± 12.31	0.499
Th (cells/μL)	262.5 (125–538)	285 (150.50-428)	0.754
Ts %	38.71 ± 11.46	44.16 ± 14.43	0.048*
Ts (cells/μL)	424 (255-591)	292.50 (187.25-431)	0.119
Th/Ts ratio	3.168 ± 5.94	0.97 ± 0.62	0.021*
B %	12.12 ± 9.91	9.55 ± 8.35	0.161
B (cells/μL)	135 ± 173.49	97.63 ± 104.82	0.193
NK %	6.33 ± 6.39	6.26 ± 5.87	0.954
NK (cells/μL)	62.10 ± 64.51	62.04 ± 85.01	0.997
Autoantibodies (n/%)			
ANA	60 (85.7%)	61 (87.1%)	0.805
Anti-dsDNA	25 (35.7%)	29 (41.4%)	0.487
Anti-nucleosome antibodies	26 (37.1%)	33 (47.1%)	0.231
Anti-histone antibodies	24 (34.3%)	27 (38.6%)	0.598
Anticardiolipin antibodies	4 (5.7%)	6 (8.6%)	0.512
Anti-β2GP-I antibodies	10 (14.3%)	9 (12.9%)	0.805
Lupus anticoagulant	34 (48.6%)	21 (30%)	0.024*

Data are mean ± SD, median (IQR), or n (%). *Bold values indicate statistical significance (p<0.05).

### 3.5 Risk factors for symptomatic ON in SLE patients

Multivariate logistic regression analysis was conducted to identify factors associated with symptomatic ON (**Table 3**). The results indicated that a history of thrombosis, LA positivity, aspirin, and the Th/Ts ratio significantly influenced the occurrence of symptomatic ON (P<0.05).

**Table 3:** Multivariable logistic regression analysis of factors for symptomatic osteonecrosis.

	OR	95% CI	P
Disease duration (months)	1.003	0.985-1.021	0.763
History of thrombosis	5.583	1.176-26.507	0.046*
Myalgia/myasthenia	355.235	0.768-16437	0.061
Neurological involvement	0.522	0.097-2.812	0.301
Glucocorticoids	7.667	0.918-64.060	0.913
Cumulative hormone use time	0.509	0.187-1.383	0.186
Aspirin	12.490	2.470-63.168	0.002*
Immunosuppressive agents	5.926	2.248-15.619	0.086
WBC	1.158	0.799-1.679	0.439
C3	57.031	0.481-6768	0.097
Lupus anticoagulant	2.204	1.101-4.409	0.032*
Total T cell percentage	1.014	0.977-1.109	0.210
Ts cell percentage	0.894	0.779-1.026	0.111
Th/Ts ratio	1.156	1.019-1.312	0.025*

*Bold values indicate statistical significance (p<0.05). OR, odds ratio; CI, confidence interval.

## 4 Discussion

ON is a recognized severe complication in patients with systemic lupus erythematosus (SLE). It leads to disability, a reduced quality of life, and increased social healthcare costs (4). Therefore, identifying high-risk factors for early ON detection in SLE patients is crucial. We observed a total of 1175 cases of SLE in our study and found the incidence rate of symptomatic ON was 6.0%. It is worth noting that the study by Oinuma et al. reported a higher incidence of ON, specifically 44%, in patients with SLE when performing MRI screening of the hip and knee (5). Our study’s lower incidence of ON could be attributed to the recruitment of patients with symptomatic ON, while the Oinuma study prospectively screened all SLE cases with MRI. Another study conducted by Nawata investigated ON incidence in SLE patients from 1986-1999 (past group) to 2000-2015 (recent group) and revealed a decrease in ON rates from 41.0% to 26.4% due to a reduced steroid dosage and the introduction of immunosuppressant agents (17). Our study investigated the incidence rate from 2012 to 2022, indicating a trend of further reduction in ON incidence in recent years, which may be attributed to early disease diagnosis and the increased use of immunosuppressive agents and biologics. In the latest study conducted within the Chinese SLE Treatment and Research Group (CSTAR), the incidence rate of ON was 2.59% during follow-ups lasting no less than 2 years (18). These differences may be due to patient demographics, the study design, and the follow-up period and warrant further large-sample multicenter studies for clarification.

In our study, SLE patients with symptomatic ON displayed an earlier onset age and a longer disease duration than the control group, aligning with previous research (7, 19). The median symptomatic ON onset time was 60 months, consistent with a mean of 5 years reported in another Chinese study (7). However, symptomatic ON likely occurs earlier than 5 years due to delayed clinical symptoms. ON involves bone tissue death, potentially from traumatic or non-traumatic events disrupting blood flow or causing osteotoxicity. The most commonly affected joints are the hip and knee. Most ON patients in this study (88.6%) experienced femoral head necrosis with bilateral involvement, in line with previous studies (7, 10, 20).

Inadequate blood supply due to thrombi presents a potential mechanism for non-traumatic avascular necrosis (AVN) of the femoral head. Consequently, we investigated thrombosis history in all SLE patients, revealing a significantly higher prevalence in the symptomatic ON group than in the control group (P<0.05). Furthermore, we observed a higher positive rate of LA in the symptomatic ON group. Logistic regression analysis confirmed LA as an SLE-related symptomatic ON risk factor. As an anti-phospholipid antibody (aPLs), LA is a significant risk factor for developing venous and arterial thrombosis (21). Previous studies also demonstrated elevated aPLs prevalence in SLE patients with ON, implying the role of aPLs-induced coagulopathy in ON pathophysiology (13, 22, 23). However, we noted no differences in anticardiolipin (aCL) and anti-β2GP-I antibodies. Some research also reported no correlation between the aPLs and the development of AVN in SLE patients adjusting for corticosteroid use (10, 24). Different types of aPLs might contribute to varying degrees. A meta-analysis on primary immune thrombocytopenia strongly suggested that LA positivity, but not aCL antibodies or anti-β2GP-I antibodies, has an increased thrombosis risk (25). Further study is essential for elucidating different aPLs roles in SLE-related ON pathogenesis.

Additionally, our study indicated a notable discrepancy in aspirin treatment, likely stemming from treatment strategies for SLE with positive LA frequently incorporating aspirin to prevent thrombosis. Anticoagulant use proved effective in preventing corticosteroid-related ON in SLE patients versus the control group (26). Thus, LA might serve as a predictor of ON occurrence. Although aspirin treatment was significantly more frequent in the symptomatic ON group, a causal relationship between aspirin and ON remains uncertain.

Measured by SLEDAI-2K, disease activity emerges as a crucial independent ON risk factor, with a higher SLEDAI-2K score indicating accelerated ON onset (27). Although our study did not detect a significant SLEDAI-2K-ON correlation, it observed notably lower complement C3 levels in symptomatic ON patients than in those without ON. Complement C3 and C4 levels often decline during high SLE disease activity (28). Abnormal differentiation of osteoclasts leads to conditions such as osteoporosis, osteosclerosis, and ON (29). Diminished C3 levels might contribute to abnormal osteoclast differentiation and potentially precipitate ON. Furthermore, low C3 is linked to complement receptor type 2 (CR2) genetic polymorphism, a membrane glycoprotein that binds C3 degradation products from complement activation. Studies identified four single nucleotide polymorphisms (SNPs) (rs3813946, rs311306, rs17615, and rs45573035) of CR2 associated with SLE-ON susceptibility (30, 31).

Previous research underscored central nervous system involvement as an ON risk factor (7, 32). Consistent with these findings, our study revealed significantly higher neurological involvement incidence in SLE patients with symptomatic ON. Moreover, symptomatic ON patients exhibited an increased incidence of myalgia/myasthenia. Statins are prevalent in high-risk patients with cerebrovascular or cardiovascular diseases and are generally considered safe. However, they sporadically induce neuromuscular side effects, accounting for approximately two-thirds of adverse events (33, 34). Our study noted a substantial rise in statin use among symptomatic ON patients (36.9% vs. 7.7%, p<0.05). Thus, myalgia/myasthenia might relate to statin use, warranting further research. Additionally, probing the central nervous system’s potential ON pathogenesis role requires additional investigation.

Univariate analysis revealed a statistical difference between both groups in corticosteroid (CS) use, cumulative hormone exposure, and immunosuppressive agents. However, multivariate analysis did not detect an increased ON risk associated with CS use. High-dose steroid pulse therapy displayed no difference between the groups. Our research suggests CS use might not be the sole ON development risk factor in SLE patients. In recent years, genetics has also been considered to play an important role in ON risk. Researchers identified a significant genetic variant on chromosome 2 (SNP rs34118383, intronic to WIPF1) associated with a 3.2 times increased ON hazard during SLE follow-up, independent of corticosteroid exposure (35). ON occurs more frequently in SLE patients than in any other rheumatic disease requiring the administration of glucocorticoids (36). This also indicates the potential involvement of additional factors within SLE leading to ON. Nevertheless, glucocorticoid-induced osteonecrosis in SLE patients should not be underestimated, and its specific pathological mechanisms still require further investigation.

In addition to hormones, immunosuppressants are vital in SLE treatment. Similar to CS, the symptomatic ON group exhibited higher immunosuppressant use than the control group. However, multivariate analysis did not establish a correlation. A substantial SLE cohort study in Korea found that patients with a cumulative corticosteroid dose of  >20 g and immunosuppressant use had a 15.44-fold increased avascular necrosis risk. Interaction analysis supported synergistic effects in AVN development (19). Conversely, another study indicated that introducing immunosuppressant agents can reduce glucocorticoid-associated osteonecrosis incidence in SLE patients (17). Therefore, balancing glucocorticoid and immunosuppressant use for ON treatment and prevention in SLE patients is challenging and requires further exploration. Research is warranted to untangle these medicines’ intricate interplay driving ON development.

Immunologic factors have been implicated in osteonecrosis pathogenesis. Lymphocytes, including T, B, and NK cells, maintain immune function; variations in these subsets can disrupt immune homeostasis. Lymphocytes also play a crucial role in the pathogenesis of femoral head symptomatic ON (37). Abnormalities of T cells, particularly CD4+ helper (Th) and CD8+ cytotoxic (Ts) subsets, are a prominent factor contributing to the exacerbation of chronic inflammation and tissue damage in SLE (38). Our study first identified a correlation between SLE patients with symptomatic ON and decreased Ts cells. Ts cells are a type of T cell that can suppress the activity of other immune cells. These cells contribute to immune response modulation, preventing excessive immune reactions and thereby maintaining immune homeostasis, reducing bone resorption and destruction, and promoting bone remodeling and metabolic balance (39). Ts cells frequently display a functional impairment in patients with SLE (40). Research by Jinhui Ma et al. also showed a higher total lymphocyte count and lower Ts cells in osteonecrosis patients (37). However, our study found no differences in total lymphocyte count and other subsets, possibly due to the small sample size. Elevated Th/Ts ratio, stemming from decreased Ts cells, was an independent symptomatic ON risk factor. A recent study also indicates that in SLE patients with EBV infection, there is a higher Th/Ts ratio than those without EBV DNA, indicating its significant role in the complications of SLE (41). The relationship between Ts cells and SLE with ON requires large-scale prospective studies for validation.

Although our study provides valuable insights into SLE with symptomatic ON, several limitations should be acknowledged. First, the sample size in our study was relatively small, which may impact the generalizability of our findings to a broader SLE population. Second, the retrospective nature of the study design introduces inherent limitations, including potential selection bias and the reliance on available medical records. Third, not all SLE patients in the control group underwent imaging examinations. As a result, it is acknowledged that asymptomatic osteonecrosis may not have been detected in some cases, which constitutes another limitation. Future prospective studies, with larger sample sizes, standardized imaging protocols, and comprehensive data collection, are warranted to further validate and extend our findings.

In conclusion, our study establishes a close connection between immunomodulatory cells, particularly Ts lymphocytes, and ON pathogenesis. Elevated Th/Ts ratio is an ON risk factor in SLE. Additionally, thrombosis history and LA elevate ON likelihood, potentially serving as ON predictors. Clinicians should remain vigilant in clinical diagnosis and treatment.

## Data availability statement

The original contributions presented in the study are included in the article/supplementary material. Further inquiries can be directed to the corresponding authors.

## Ethics statement

This study received approval from the ethical committee of Shanxi Bethune Hospital. The studies were conducted in accordance with the local legislation and institutional requirements. The participants provided their written informed consent to participate in this study. Written informed consent was obtained from the individual(s) for the publication of any potentially identifiable images or data included in this article.

## Author contributions

RH: Writing – original draft. JL: Data curation, Investigation, Writing – original draft. DX: Investigation, Writing – original draft. YJ: Funding acquisition, Writing – original draft. LM: Investigation, Writing – original draft. QG: Supervision, Writing – original draft. LZ: Supervision, Writing – review & editing. KX: Writing – review & editing, Supervision.
